# Waterless Dyeing and In Vitro Toxicological Properties of Biocolorants from *Cortinarius sanguineus*

**DOI:** 10.3390/jof8111129

**Published:** 2022-10-26

**Authors:** Mikko Herrala, Johanna Yli-Öyrä, Anjaína Fernandes de Albuquerque, Natália Oliveira de Farias, Daniel Alexandre Morales, Riikka Räisänen, Harold S. Freeman, Gisela Aragão Umbuzeiro, Jaana Rysä

**Affiliations:** 1 School of Pharmacy, University of Eastern Finland, 70210 Kuopio, Finland; 2 School of Technology, University of Campinas, 13484-332 Limeira, São Paulo, Brazil; 3 Craft Studies, University of Helsinki, 00014 Helsinki, Finland; 4 Wilson College of Textiles, North Carolina State University, Raleigh, NC 27695-8301, USA

**Keywords:** bloodred webcap, dermocybin, dermorubin, emodin, fungal colorants, supercritical CO_2_, toxicity, waterless dyeing

## Abstract

As a part of an ongoing interest in identifying environmentally friendly alternatives to synthetic dyes and in using liquid CO_2_ as a waterless medium for applying the resulting colorants to textiles, our attention turned to yellow-to-red biocolorants produced by *Cortinarius sanguineus* fungus. The three principal target anthraquinone colorants (emodin, dermocybin, and dermorubin) were isolated from the fungal bodies using a liquid–liquid separation method and characterized using 700 MHz NMR and high-resolution mass spectral analyses. Following structure confirmations, the three colorants were examined for dyeing synthetic polyester (PET) textile fibers in supercritical CO_2_. We found that all three biocolorants were suitable for dyeing PET fibers using this technology, and our attention then turned to determining their toxicological properties. As emodin has shown mutagenic potential in previous studies, we concentrated our present toxicity studies on dermocybin and dermorubin. Both colorants were non-mutagenic, presented low cellular toxicity, and did not induce skin sensitization. Taken together, our results indicate that dermocybin and dermorubin possess the technical and toxicological properties needed for consideration as synthetic dye alternatives under conditions that are free of wastewater production.

## 1. Introduction

The origin and safety of colors is rarely considered by the average consumer, even though the color itself is one of the most important factors taken into account when purchasing objects [1]. Even so, the global market for dyes and pigments was valued at USD 32.9 billion in 2020, with a predicted annual growth of over 5% [2]. Because of their purpose of use, synthetic colorants are chemically very stable and can persist for long periods when they enter the environment. Even low concentrations of dyes in water systems can reduce the penetration of sunlight and, as a result, inhibit photosynthesis, causing oxygen deprivation and toxic effects to aquatic organisms from many trophic levels [3,4,5,6]. It has also been studied that certain azo dyes—the largest class of synthetic colorants—pose occupational health risks, such as allergic sensitization and a higher prevalence of some cancers [7,8,9]. Therefore, it is not surprising that there is an increasing interest in developing safer and more sustainable dyes and dyeing methods [10,11,12,13]. Even though most of the dyes produced worldwide are used by the textile industry [2], the development of eco-friendly dyes would also benefit the development of substrates such as biodegradable packaging [14].

**Figure 1:**
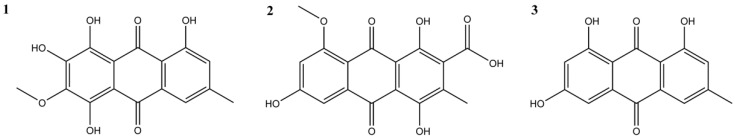
The chemical structures of the dyes present in *Cortinarius sanguineus*: (**1**) dermocybin, (**2**) dermorubin and (**3**) emodin.

Anthraquinones comprise the second largest class of commercial dyes next to azo dyes. They have a common 9,10-anthracenedione backbone, which is not a dye, but the addition of auxochromes, such as hydroxyl, amino, or nitro groups, produces dyes varying in color from yellow and red to blue [15]. The manufacturing of anthraquinones is expensive and demands the use of problematic chemicals, which has diminished their importance [16,17]. In nature, anthraquinones are produced by several plants, fungi, and other organisms [18] and they possess antioxidative properties in addition to their color [19]. An example of an anthraquinone producer is bloodred webcap (*Cortinarius sanguineus*), a common fungus found in the coniferous forests of North America, Northern Europe, and the British Isles. It produces yellow-to-red anthraquinone dyes—mainly emodin, dermocybin, and dermorubin, which represent approximately 6% of its dry weight [17]. The colorant proportions depend on the geographical origin: the color composition of the Finnish bloodred webcap is approximately 63% emodin, 31% dermocybin, and 4% dermorubin [20,21]. Their structures are presented in Figure 1.

The safety of naturally occurring anthraquinones as a group has been reviewed by Brown [22], Sendelbach [23], and Shukla et al. [19]. Based on these reviews, anthraquinones exhibit toxicological diversity in addition to their biological and chemical variety and, therefore, it is of interest to study the toxicity of individual dyes. Of the *C. sanguineus* dyes, emodin is the most studied compound. It has mutagenic and skin sensitizing potential, and it undergoes metabolic activation via cytochrome P450 (CYP) enzymes to 2-hydroxy-emodin. This metabolite is a known direct-acting mutagen [24] but, on the other hand, it can also suppress cancer growth and scavenge reactive oxygen species [25]. However, nothing is known about the toxicity of dermocybin and dermorubin as far as we are aware.

Synthetic anthraquinone dyes are widely used in the coloration of natural and synthetic fibers, and most of their commercial use involves dyeing from water. The volume of water required to dye one kilogram of fabric approaches 150 L. Consequently, significant amounts of wastewater are produced in these processes, and they require treatment prior to discharge that could potentially harm human health or the environment. In addition, with fresh water being a precious commodity worldwide, approaches to conserving its reserves are of universal interest [26,27]. This vision has sparked the development of waterless media for textile dyeing, including the liquid CO_2_ technology first reported by Saus et al. [28]. The best results were obtained when using the family of hydrophobic disperse dyes in the coloration of polyester fabric [28,29]. Further, the fabric is completely dry when it exits the supercritical-CO_2_ dyeing process, eliminating the need for rinsing it with water and the high energy usage associated with the traditional dyeing process, involving the heating of aqueous media and the final drying of the textile.

The anthraquinone dyes from blood-red webcap are able to dye polyester fibers in aqueous media, as studied by Räisänen et al. [30]. Based on this information, a key goal of our present work was to determine the utility of supercritical-CO_2_ media for applying these natural dyes, or biocolorants, to textiles. This would provide not only biocolorants for textiles but also a waterless dyeing alternative. First tests have been conducted with emodin, and the successful results were published recently [27]. Bearing in mind that a natural origin does not guarantee the safety of these biocolorants, we also set out to establish the previously unknown toxicological properties of dermocybin and dermorubin. Acknowledging the need to reduce and find alternatives for animal testing, we used in vitro methods for assessing their cytotoxicity, skin sensitization potential, and mutagenicity.

## 2. Materials and Methods

### 2.1. Analytical Methods

The dyes were extracted from the blood-red webcap fungal bodies and the anthraquinones were separated using multiple liquid–liquid partition as described before [20,21]. No further purification was undertaken on the collected fractions. The purity and chemical structures of the compounds were confirmed by high-performance liquid chromatography coupled with diode-array detection and electrospray ionization tandem mass spectrometry (HPLC-DAD-MS) and nuclear magnetic resonance (NMR) spectroscopy (^13^C and ^1^H).

HPLC-DAD was carried out using a C18 (XBridge) 5 µm, 3.0 × 100 mm column. The mobile phase was an acetonitrile and 0.1% formic acid (Thermo Fisher Scientific, Waltham, MA, USA) gradient solution with a flow rate 1.0 mL/min and temperature of 20 °C (conditions presented in Supplementary Materials, Table S1). Recorded absorption wavelengths were 480 nm for dermorubin and 487 nm for dermocybin.

MS analysis was carried out using a high-resolution mass spectrometer (HRMS, Thermo Fisher Scientific Exactive Plus MS, a benchtop full-scan Orbitrap mass spectrometer) using heated electrospray ionization (HESI). Samples were diluted in acetone and analyzed via flow injection into the mass spectrometer at a flow rate of 200 µL/min. The mobile phase was 90% acetonitrile with 0.1% formic acid and 10% water with 0.1% formic acid (all chemicals from Thermo Fisher Scientific, Waltham, MA, USA). The mass spectrometer was operated in the positive ion mode.

The NMR spectra of dermocybin and dermorubin were recorded on a Bruker Avance 700 MHz NMR spectrometer equipped with an Oxford Narrow Bore Magnet, a 5 mm QXI SB probe, and HP XW Host Workstation. Before analysis, samples were dissolved in dimethyl sulfoxide-d_6_ (DMSO-d_6_). ^1^H-NMR and ^13^C-NMR chemical shifts (δ) are reported in ppm relative to the tetramethylsilane (TMS) signal. Topspin software from Bruker was used for data processing.

#### 2.1.1. Visible Absorption Maxima

Emodin (1,3,8-trihydroxy-6-methyl-9,10-anthraquinone): 441 nm;
Dermorubin (1,3,4,5-tetrahydroxy-2-methoxy-7-methyl-9,10-anthraquinone): 480 nm;
Dermocybin (1,4,6-trihydroxy-8-methoxy-3-methyl-9,10-anthraquinone-2-carboxylic acid): 487 nm.

#### 2.1.2. HRMS

Emodin: C₁₅H₁₀O₅; M_calc_ = 271.0606 [M + H]^+^; M_expl_ = 271.0604;
Dermocybin: C_16_H_13_O_7_; M_calc_ = 317.0656 [M + H]^+^, M_expl_ = 317.0650;
Dermorubin: C_17_H_13_O_8_; M_calc_ = 345.0605 [M + H]^+^; M_expl_ = 345.0598.

#### 2.1.3. ^1^H-NMR Spectra (ppm, DMF-d_7_)

Emodin: δ12.00 (s, 1H), 11.90 (s, 1H), 11.30 (s, 1H), 7.45 (s, 1H), 7.10 (s, 1H), 7.05 (s, 1H), 6.55 (s, 1H), 2.55 (s, 3H);
Dermocybin: δ13.96 (s, 1H), 12.09 (s, 1H), 8.02 (s, 1H), 7.65 (s, 1H), 7.20 (s, 1H), 4.01 (s, 3H), 2.50 (s, 3H);
Dermorubin: δ13.80 (s, 1H), 12.99 (s, 1H), 11.30 (bs, 1H), 7.46 (s, 1H), 6.81 (s, 1H), 3.95 (s, 3H), 2.45 (s, 3H).

#### 2.1.4. ^13^C-NMR Spectra (ppm, DMF-d_7_)

Emodin: δ190.5, 181.6, 166.0, 164.9, 161.8, 148.6, 135.4, 133.1, 124.5, 120.8, 113.6, 109.3, 109.2, 108.3, 21.9;
Dermocybin: δ190.0, 184.7, 161.8, 149.3, 146.1, 141.9, 139.2, 133.8, 123.8, 120.1, 114.1, 108.6, 60.8, 21.4;
Dermorubin: δ187.2, 185.1, 167.0, 165.0, 155.1, 152.5, 136.8, 135.7, 113.2, 112.9, 107.5, 56.7, 12.5.

### 2.2. Dyeing

Dyeing was conducted in a Pressure Products Industries, Inc. (PPI) reaction vessel, equipped with a mechanical stirrer. For each dyeing, a 2.5 g fabric sample was sewn end to end and slid over the cylindrical sample holder. Powdered dye (12.5–25 mg, 0.5–1.0% of the weight on the fiber, o.w.f.) was placed on a stainless-steel mesh at the bottom of the sample holder. The sample holder containing dye and fabric was placed in the dyeing chamber and the vessel was closed tightly. Liquid CO_2_ was run into the dyeing vessel through a stainless-steel pipe and stirring and heating of the jacketed vessel were initiated. Dyeing was conducted for 30 min at 120 °C and 5000 psi. The dyeing system was evacuated, allowed to cool over 1 h, and the vessel was opened to remove the dyed fabric. CIE L*a*b* values were measured with a Datacolor SF600 computer color-matching instrument. A D65 light source was adopted with an observation angle of 10°. L* depicts the lightness/darkness level of the dyed textiles, where the scale is 0 (black/dark) to 100 (white/light). Similarly, a* depicts the greenness/redness level of the fabric, where the more negative the a* value, the greener the shade, and the more positive the a* value, the redder the shade. Further, blue fabrics are characterized by a negative b* value and yellow fabrics give a positive b* value.

The quality of dye penetration into synthetic polyester (PET) fibers was studied according to the American Association of Textile Chemists and Colorists (AATCC) TM8 rubbing (crock) fastness test [31], which is similar to the ISO 105-X12 standard. In this test, a white crocking square is rubbed against the dyed fabric for the specified number of times (10) and then analyzed using a color transfer scale 1–5, where 1 expresses great color transfer and 5 indicates no color transfer.

The stability of the dyed fabric against color removal during washing (wash fastness) was assessed using a modified version of the AATCC TM61 Method 2A wash test [32], in which 8 steel balls were used instead of 16 because of the smaller fabric sample size (5 cm × 10 cm vs. 5 cm × 20 cm). This test simulates a home machine-washing process, and it is partly equivalent to the ISO 105-C06 standard. The color difference, ∆E, of washed fabric compared to unwashed fabric was analyzed using an X-Rite spectrophotometer (Grand Rapids, MI, USA), following the equation $\Delta E=\sqrt{{\left(\Delta L^{*}\right)}^{2}+{\left(\Delta a^{*}\right)}^{2}+{\left(\Delta b^{*}\right)}^{2}}$, where the ∆ values represent the differences between the washed and unwashed fabrics.

### 2.3. Cell Culture and Exposures

Hepatic HepG2 and human acute monocytic leukemia THP-1 cell lines were used to determine the cellular toxicity of the dyes. HepG2 cells were cultured in Dulbecco’s modified Eagle medium containing 1.0 g/L glucose (DMEM, Gibco, Paisley, UK), supplemented with 10% fetal bovine serum (FBS, Merck, Darmstadt, Germany), 1% L-glutamine (Gibco, Paisley, UK), 100 U/mL penicillin and 100 µg/mL streptomycin (Lonza Group Ltd., Basel, Switzerland). The cells were detached after trypsinization (0.05% Trypsin-EDTA (Gibco, Paisley, UK) in Dulbecco’s phosphate buffer saline (DPBS)), and 100,000 cells in 500 µL of medium per well were seeded in 48-well plates (Thermo Fischer Scientific, Waltham, MA, USA) 24 h prior to exposure.

THP-1 cells were cultured in RPMI-1640 medium (HEPES, no glutamine, Gibco, Paisley, UK), supplemented with 10% FBS performance plus (Gibco, Paisley, UK), 1% L-glutamine (Gibco, Paisley, UK), and 100 U/mL penicillin and 100 μg/mL streptomycin (Lonza Group Ltd.). In the experiments, cells were seeded on 48-well plates, 175,000 cells in 500 µL of medium per well. The cells were differentiated into macrophages with phorbol 12-myristate 13-acetate (PMA, Sigma Aldrich, Darmstadt, Germany). Differentiation and adherence were initiated by adding 50 nM PMA into the cell-culture medium and changing fresh PMA medium on the following day. After 48 h pre-treatment, the differentiation medium was replaced with exposure medium.

All cell cultures were maintained in cell-culture flasks with a 75 cm^2^ cell-culture area (VWR, Radnor, PA, USA) in a humidified incubator (MCO-170M-PE, PHC Corporation, PHCbi, Tokyo, Japan), with 5% CO_2_ and a temperature of 37 °C.

In cytotoxicity, oxidative stress, and skin sensitization studies, the tested dyes were diluted in dimethyl sulfoxide (DMSO, Sigma Aldrich, St. Louis, MO, USA) at their limit of solubility. Exposure concentrations were diluted with culture medium, 0.03–10 µg/mL for dermorubin and 0.035–7 µg/mL for dermocybin. The highest concentration was chosen so that the highest DMSO dose would be 0.1%. Accordingly, 0.1% DMSO was used as a negative control. However, in the skin sensitization assay, the highest doses were 100 µg/mL for dermorubin and 70 µg/mL for dermocybin, in which case the highest DMSO concentration was 1.0%, which was also the concentration of the negative control. The cells were exposed to the colorants for 24 h, except in the skin sensitization assay where the exposure time was 48 h. Each test was conducted 3–4 times, and there were 3–4 parallel samples per concentration. Positive controls were as follows: 0.1% Triton X-100 (Sigma Aldrich, St. Louis, MO, USA) was used in the cell viability, cytotoxicity, and the combined reactive oxygen species (ROS) production–viability assays. A 200 µM concentration of menadione (Sigma Aldrich, St. Louis, MO, USA) was utilized in the oxidative stress studies, and 15.625–250 µM ethylene glycol dimethacrylate (EGDMA, acCELLerate GmbH, Hamburg, Germany) in the skin sensitization assay.

### 2.4. Cell Viability and Cytotoxicity

After 24 h exposure, cell viability was assessed with an MTT (3-(4,5-dimethylthiazol-2-yl)-2,5-diphenyltetrazolium bromide) assay as described earlier in Pesonen et al. [33]. In addition to the MTT assay, the lactate dehydrogenase (LDH) assay was used to assess both apoptotic and necrotic cell death [34]. LDH release was measured from media collected from 48-well plates after 24 h dye exposure. The LDH assay mixture (Sigma Aldrich, St. Louis, MO, USA) was prepared and added into the well plate and the cells were incubated for 20 min in the dark at room temperature before measuring the absorbance with a microplate reader (Hidex Sense 425-301 microplate reader, Hidex, Turku, Finland).

### 2.5. Oxidative Stress Responses

#### 2.5.1. Reactive Oxygen Species Production and Cell Viability

ROS production was measured with 2′,7′-dichlorodihydrofluorescein diacetate (H_2_DCFDA, Sigma Aldrich, St. Louis, MO, USA) after 24 h dye exposure as described by Loikkanen et al. [35]. Immediately after the DCF assay, cell viability was measured using propidium iodide (PI, 50 μM, Sigma Aldrich, St. Louis, MO, USA) and digitonin (160 μM, Calbiochem, Darmstadt, Germany), which are used to measure necrotic cell death. Cell viability was expressed in relation to maximum PI value using viability formula $viability=100-\frac{F-blank_{1}}{F_{max}-blank_{2}}\times 100\%$, where F is fluorescence, blank_1_ is the blank after PI addition, F_max_ is fluorescence and blank_2_ is the blank after digitonin addition. Maximum fluorescence value represents the total number of cells in each well. Thereafter, for each exposure concentration, the relation of DCF fluorescence to the cell amount was calculated.

#### 2.5.2. Cytosolic and Mitochondrial Superoxide Production

Cytoplasmic superoxide (O_2_^•-^) production was measured after 24 h dye exposure using a fluorescent probe dihydroxy ethidium (DHE, Fluka Biochemica, Buchs, Switzerland) as described by Höytö et al. [36]. Furthermore, MitoSOX^TM^ Red mitochondrial O_2_^•-^ indicator (Molecular Probes, Invitrogen, Paisley, UK) was used to measure mitochondrial O_2_^•-^ production after 24 h exposure to dyes.

### 2.6. Skin Sensitization

Skin sensitization potential was measured with the commercial instaCELL^TM^ KeratinoSens^TM^ assay kit (acCELLerate GmbH) based on a luciferase Nrf2 gene-activation test [37]. Briefly, assay-ready KeratinoSens cells were thawed and seeded onto 96-well plates. After 24 h incubation in a humidified incubator (PHC Corporation), with 5% CO_2_ and a temperature of 37 °C, the cells were exposed. After 48 h exposure, 20 µL of resazurin was added to each well to define cell viability. After a 4 h incubation at 37 °C, fluorescence was measured using a fluorometer (Hidex). Immediately after measurement, the medium was removed, and the cells were washed once with DPBS and then 50 µL of DPBS and 50 µL of One-GloTM reagent was added per well. After 20 min incubation at room temperature in the dark, luminescence was measured with an integration time of 1 s/well. Induction is considered to have occurred if gene activation was enhanced by 50% (i.e., 1.5 times greater than the control activation).

### 2.7. Mutagenicity

The mutagenicity was evaluated using a miniaturized protocol of the *Salmonella*/microsome mutagenicity assay, the microplate agar (MPA) described by Zwarg et al. [38]. Dyes were tested with five strains of *Salmonella enterica* serovar *Typhimurium* in a tiered approach as recommended by Umbuzeiro and others [39], starting with YG1041 followed by TA97a, TA98, and TA100 in concentration-response experiments. The TA1537 strain was also included because it is known to be sensitive to anthraquinone compounds, including several natural dyes, as reported by Brown and Brown [40]. Tests were performed in the absence and presence of the metabolic activation system provided by Aroclor 1254-induced Sprague Dawley rat liver S9 mix (MolTox, Boone, NC, USA) with the required cofactors at 5% of S9 in the mixture [41]. TA1537 was also tested with 10% of S9 as this condition is known to be optimal for the activation of anthraquinones [40]. The tested dyes were diluted in DMSO at the limit of their solubility. Dermocybin concentrations ranged from 1.67 to 500 ng/µL, and dermorubin concentrations from 3.33 to 1000 ng/µL. DMSO was used as a negative control and positive controls with and without metabolic activation were also included.

### 2.8. Statistical Analysis

Data from in vitro toxicity experiments were analyzed with analysis of variance (ANOVA) followed by Tukey’s post hoc test. GraphPad Prism version 5.03 for Windows (GraphPad Prism Inc., San Diego, CA, USA) and the SPSS software for Windows release 27 (SPSS Inc., Chicago, IL, USA) were used for statistical analyses. Results are expressed as mean ± standard error of the mean (SEM). Differences where *p* < 0.05 were considered as statistically significant.

The experimental data from mutagenicity were analyzed with the Salanal program (Integrated Laboratory Systems, Research Triangle Park, NC, USA) using ANOVA followed by a linear regression. The sample was considered positive when both the ANOVA and linear regression provided significant responses (*p* < 0.05). Experiments were only considered valid when the cell viability and positive controls responded accordingly [38].

## 3. Results

### 3.1. Chemical Characterization

According to the HPLC-DAD analysis, the purity of emodin was 98.3%, dermocybin 99.7%, and the dermorubin sample contained 98.1% dermorubin and 1.6% 5-Cl-dermorubin. Due to its purity of over 98%, the sample is considered essentially as dermorubin. The experimental MS values were very close to those calculated, with each experimental value within at least 0.0007 *m*/*z* unit of the calculated value of the parent derived ion. The experimental proton and carbon NMR shifts for the two dyes are also presented, all of which are consistent with the molecular structures.

### 3.2. Dyeing Studies

Results from dyeing PET fabric in supercritical CO_2_ (Sc-CO_2_) are illustrated in Table 1 for 0.5% (*w*/*w*) dye application. Emodin, dermocybin, and dermorubin can be applied from this medium, with dermocybin giving the deepest shade at L* = 71.67. PET also undergoes a significant color shift when emodin is replaced by dermocybin, owing to an increase in red character (a* = 23.33 vs. −1.54) and a decrease in yellow character (b* = 48.24 vs. 67.94). While dermorubin and dermocybin give similar colors on PET, the shade depth from dermorubin is clearly weaker, probably due to a lower CO_2_ solubility arising from the carboxylic acid group in its structure. Thus, esterification of the carboxyl group using 2-methoxyethanol (HOCH_2_CH_2_OCH_3_) enhanced the dye uptake, giving a deeper shade even at 0.25% (*w*/*w*) [42]. Dyeing with dermorubin also facilitated the optimization of CO_2_ pressure, as poor dye uptake occurred on PET in the 3500–4000 psi region.

Results obtained from color fastness tests are summarized in Table 2. The absence of surface dye was indicated in the lack of dye on the crocking fabric in the rubbing fastness test, and a rating of 5 on a scale of 1 to 5 (high color transfer–no color transfer) was observed for each of the three dyes on PET, indicating excellent dye penetration. Furthermore, from Table 2, it can be seen that emodin and dermocybin gave the best results for color change/loss indicated from a value near zero for ∆E and color change value of 4 (gray scale rating), while dermorubin received slightly more change in color, indicated from a greater value of ∆E and lower value of color change (gray scale rating 3.5).

### 3.3. Cell Viability and Cytotoxicity

**Figure 2:**
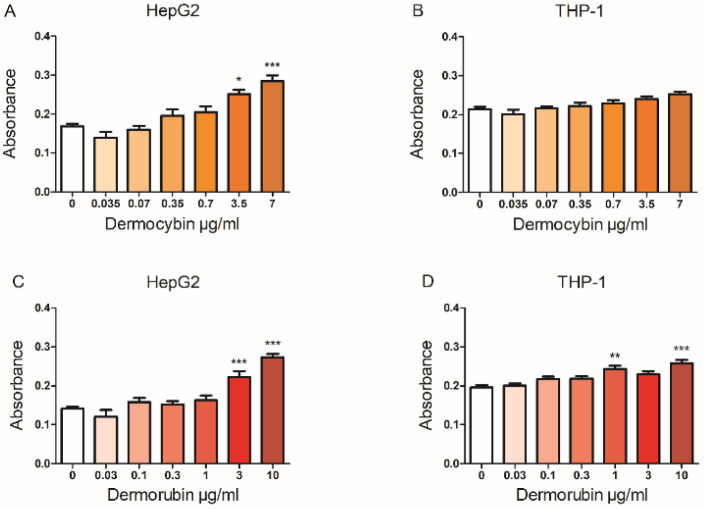
Lactate dehydrogenase test results. (**A**) and (**B**) represent dermocybin, (**C**) and (**D**) represent dermorubin. Data are shown as absorbance mean ± SEM, * *p* < 0.05, ** *p* < 0.01, and *** *p* < 0.001, *n* = 3.

Dermocybin increased the LDH release in HepG2 cells at the two highest concentrations (*p* < 0.05 for 3.5 µg/mL and *p* < 0.001 for 7 µg/mL, Figure 2) but had no effect in THP-1 cells. Dermorubin, on the other hand, evoked cytotoxicity in HepG2 cells at 3 µg/mL (*p* < 0.001) and 10 µg/mL concentrations (*p* < 0.001), and in THP-1 cells at 1 µg/mL (*p* < 0.001) and 10 µg/mL (*p* < 0.01).

**Figure 3:**
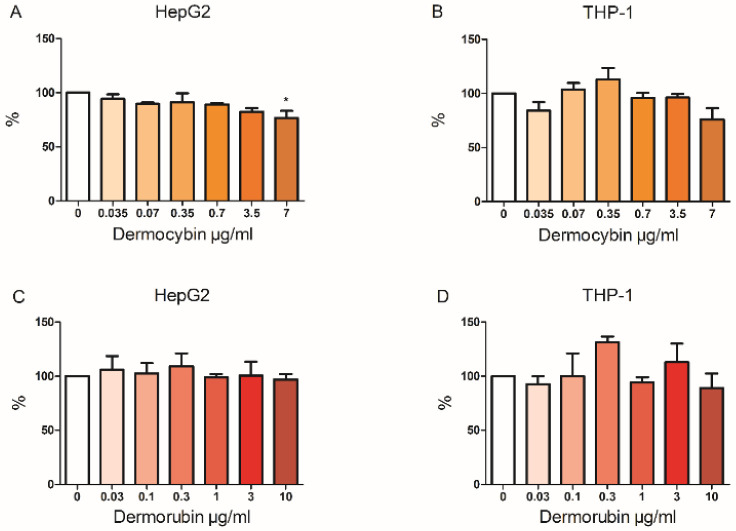
Cell viability measured with MTT after 24 h exposure to dermocybin and dermorubin. (**A**) and (**B**) represent dermocybin, (**C**) and (**D**) represent dermorubin. Data are shown as % of control ± SEM, * *p* < 0.05, *n* = 3.

Dermorubin had no effect on cell viability in the HepG2 or THP-1 cells in the MTT assay (Figure 3). Dermocybin decreased the HepG2 cell viability at the highest 7 µg/mL concentration to 76.7% as compared to control cells (*p* < 0.05) but no effects were observed in THP-1 cells. In addition, the cell viability of HepG2 and THP-1 cells was not affected in the PI–digitonin assay (Supplementary Materials, Figure S1).

### 3.4. Oxidative Stress Responses

**Figure 4:**
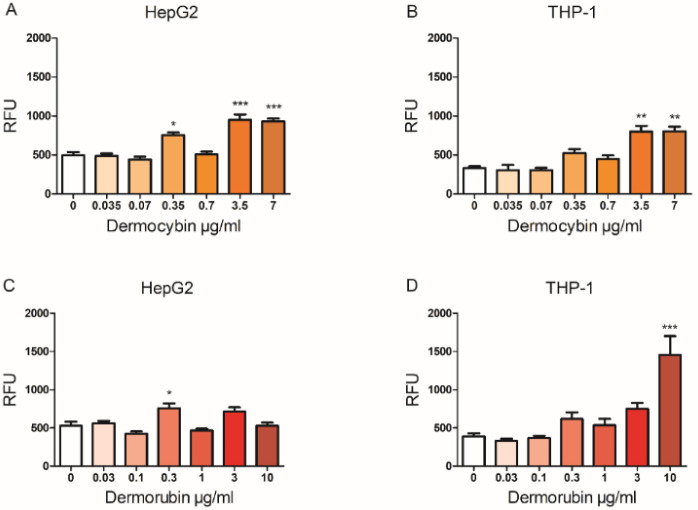
The effect of dermocybin and dermorubin exposure on mitochondrial superoxide production in HepG2 and THP-1 cells. (**A**) and (**B**) represent dermocybin, (**C**) and (**D**) represent dermorubin. RFU = relative fluorescence unit. Data are shown as fluorescence mean ± SEM, * *p* < 0.05, ** *p* < 0.01 and *** *p* < 0.001, *n* = 3–4.

Dermocybin increased the mitochondrial superoxide production in HepG2 cells at three concentrations: 0.035 µg/mL (*p* < 0.05), 3.5, and 7 µg/mL (*p* < 0.001). A similar increase at the two highest concentrations was also observed in THP-1 cells (*p* < 0.01), as shown in Figure 4.

Dermorubin caused a nearly threefold increase in mitochondrial superoxide production in THP-1 cells at the highest dose (*p* < 0.001). In addition, a minor mitochondrial superoxide increase was observed at the 0.3 µg/mL dose in HepG2 cells (*p* < 0.05). However, no changes were observed in cytosolic superoxide production or general ROS levels after 24 h exposure to dermocybin or dermorubin in either cell line (Supplementary Materials, Figure S2).

### 3.5. Skin Sensitization

**Figure 5:**
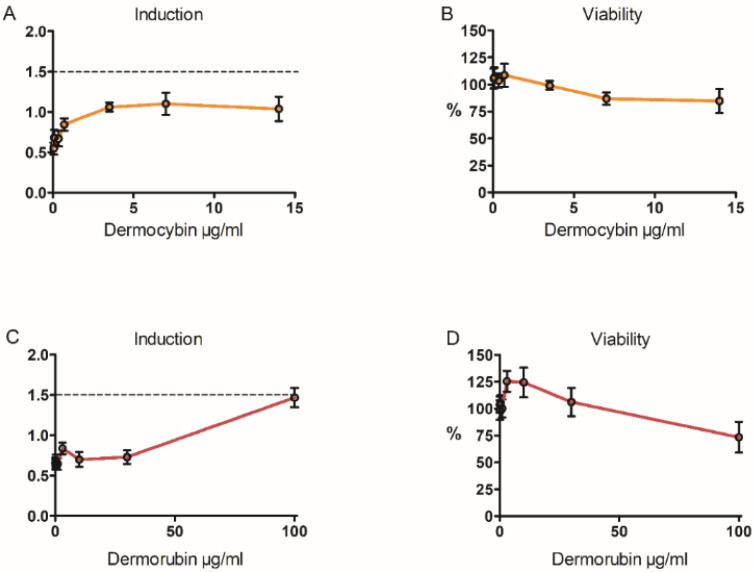
Results of the skin sensitization assay. In figures (**A**) and (**C**), the reporter gene induction compared to control is shown. A 1.5-fold increase is defined as the limit of induction (dotted line). In figures (**B**) and (**D**), keratinocyte viability compared to control is presented, *n* = 4.

Dermorubin and dermocybin were assessed for their skin sensitization potential using the KeratinoSens assay. Dermocybin precipitated at concentrations of 28–70 µg/mL, causing total cell death. Therefore, those results were omitted and only dermocybin concentrations up to 14 µg/mL are presented in Figure 5. Cell viability was measured with resazurin before a reporter gene activation assay, and the results were in line with others: a small reduction in viability was seen at higher exposures. Based on the results of the KeratinoSens reporter gene activation assay, neither dermocybin nor dermorubin are skin sensitizers, as the 1.5-fold induction limit was not exceeded at any dose, even though the level of induction caused by 100 µg/mL dermorubin was near to the limit, being 1.47 as compared to control.

### 3.6. Mutagenicity

Dermocybin and dermorubin were tested using the tiered approach introduced by Umbuzeiro et al. [39]. Dye concentrations were decided based on the maximum solubility in DMSO. Neither dye induced mutagenic effects with or without metabolic activation with S9 in *S. Typhimurium* strains YG1041, TA98, TA100, TA97a, and TA1537 used for testing. Because anthraquinones may require more concentrated S9, they were tested with TA1537, the strain most sensitive to some natural anthraquinones, and also using 10% of S9, and no mutagenic effects were obtained (Table 3 and Table 4).

## 4. Discussion

Anthraquinones are the second most widely used group of dyes by the textile industry. In fungi, they exist as complex mixtures. For example, fourteen different derivatives have been identified from *Cortinarius sanguineus*, with one other compound remaining unidentified [43]. By adopting a liquid–liquid separation method, which is based on the compounds’ pH-dependent solubilities between organic and aqueous phases, the main anthraquinone derivatives were obtained at high purity. This is the first time that the complete proton and carbon NMR spectra for dermocybin and dermorubin have been reported. The emodin proton NMR spectrum has been reported previously by Danielsen and coworkers [44], which included signals arising from emodin’s -OH protons when chloroform-d_1_ was used as the solvent. However, because these compounds are obtained from seldom-studied fungi, almost nothing was known about either their dyeing properties in waterless media or their toxicological properties.

The structural similarities between these biocolorants and the well-known hydrophobic synthetic anthraquinone disperse dyes indicated that they would be logical options for dyeing PET fibers. This was confirmed in our study for all three anthraquinones. Moreover, our previous studies revealed that high-temperature disperse dyeing technique results in bright colors with very high colorfastness towards washing, rubbing, and light, in PET fabric [30], and similar qualities are expected after Sc-CO_2_ applications. These expectations were fulfilled as our results with emodin, dermocybin, and dermorubin indicated excellent dye penetration in PET fibers. Furthermore, emodin and dermocybin gave the best results for color change/loss, while slightly more change in color was observed with dermorubin. Since the washing medium was slightly alkaline, it is possible that the enhanced dye removal is due to the conversion of some of dermorubin’s CO_2_H groups to dye molecules with water-solubilizing CO_2_ groups. Further, the present natural dyes join a small group of synthetic anthraquinone dyes known to dye PET in Sc-CO_2_ [45].

In our toxicological studies, we demonstrated that dermocybin and dermorubin are not mutagenic. The studies were conducted using the MPA protocol, a cost-effective mutagenicity test whose sensitivity matches the regular Ames test but requires fewer reagents and less labor [38]. Five *S. Typhimurium* strains with and without metabolic activation were tested over a wide range of colorant concentrations, and no mutagenic effects were observed. Supporting our results, dermocybin was listed as non-mutagenic in the review by Brown [22].

Furthermore, dermocybin and dermorubin caused no consistent, dose-dependent toxicity in cell viability assays, indicating a low level of toxicity. We observed increased LDH release connected to both colorants in HepG2 cells, whereas THP-1 cells were affected only by dermorubin. However, no decrease in cell viability was observed in the PI-digitonin assay or in the MTT test, except for the slight reduction in HepG2 viability at the highest dermocybin concentration. In general, the effect of natural anthraquinone dyes on cell viability has been scarcely studied; the only previous report we found claimed that rhein, another plant-derived anthraquinone, caused a clear dose-dependent reduction in cell viability using MTT and neutral red uptake tests in rat primary hepatocytes [46]. In contrast to that publication, our results do not raise concern about the cytotoxicity of dermocybin or dermorubin.

When assessing oxidative stress markers, the mitochondrial superoxide production increased at the highest dermocybin doses in both cell lines and, in THP-1 cells, also with dermorubin. Excessive amounts of mitochondrial ROS could impair the respiratory chain reactions and subsequently damage mitochondrial DNA, which could ultimately trigger cellular apoptosis [47,48]. However, no increase in cytosolic superoxide or cellular ROS production was detected, and the increased mitochondrial superoxide production did not manifest as reduced cell viability. Interestingly, in addition to the radical-scavenging properties of emodin [25], purpurin, a red natural anthraquinone dye, has been shown to reduce oxidative stress [49]. Our results expand the understanding of the oxidative properties of anthraquinones, which could also be beneficial in industrial applications.

Dermocybin and dermorubin were not skin sensitizers according to the KeratinoSens assay. This assay has been validated in collaboration with the European Centre for the Validation of Alternative Methods ECVAM for in vitro skin-sensitization testing [50]. Additionally, Kim et al. [51] have compared the skin sensitization potential of carbon nanotubes with KeratinoSens in parallel with an in vivo local lymph node assay, achieving results in line with each other. Although our results provide evidence of dermocybin and dermorubin as being non-sensitizers, an integrated approach is recommended according to OECD when using in vitro methods [37].

The cellular responses observed in our study were seen only at the highest colorant concentrations. In the dye liquor, the concentration of dermocybin and dermorubin is estimated to be 0.5 mg/mL [17] when 1% (o.w.f.) concentration in 1:20 fabric-to-liquor ratio is used, which is higher than the level we were able to test in the present study. In addition, our results are applicable only to individual pure dyes. Additional testing with a *Cortinarius sanguineus* extract containing a mixture of anthraquinones is needed because the toxicity of a mixture can differ from that of single dyes. Small-scale dyers and craft enthusiasts will become exposed to these mixtures, because the *Cortinarius* extracts are normally used as such. Secondly, further studies of mitochondrial function would be helpful in clarifying the reason behind the increased mitochondrial superoxide production: persistent oxidative stress could cause chronic inflammation, evoking a diverse range of health outcomes. Additionally, as oxidative stress could cause DNA or chromosomal damage, it would be beneficial to further evaluate their genotoxic effects to clarify these potential risks.

In conclusion, it has been established that anthraquinone biocolorants derived from the fungus *C. sanguineus* are suitable for dyeing textile fibers using supercritical carbon dioxide—a waterless medium. This waste minimization through source reduction technology circumvents wastewater formation and a traditional energy-intensive fabric drying step. The coloration process was extended to toxicological studies, wherein it was found that dermocybin and dermorubin were not mutagenic, presented low cellular toxicity, and had no skin sensitization potential, making them good candidates for dyeing textiles. However, more research will be needed to assess their long-term toxicity and that of mixtures, as well as their ecotoxicological impact. Altogether these results contribute significantly to the current body of knowledge related to natural anthraquinone dyes and highlight the versatile applicability of fungi in the dyeing process.

## Figures and Tables

**Table 1:** CIE Lab color values obtained from polyester dyed with blood-red webcap dyes in Sc-CO_2_.

Natural Dye	Dyed Fabric Samples	L*	a*	b*
Emodin	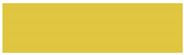	79.35	−1.54	67.94
Dermocybin	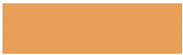	71.61	23.33	48.24
Dermorubin	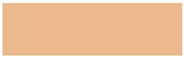	77.05	8.95	33.08

**Table 2:** Wash fastness (∆E, color change and staining) and rubbing fastness assessment results obtained from polyester fabric dyed with blood-red webcap dyes in Sc-CO_2_.

Natural Dye	Wash Fastness			Rubbing ^d^
	∆E ^a^	Color Change ^b^	Staining ^c^	
Emodin	0.83	4.0	5.0	5.0
Dermocybin	0.66	4.0	5.0	5.0
Dermorubin	1.31	3.5	5.0	5.0

^a^ Color difference values (the smaller the value, the less dye removed); ^b^ Color change expressed as a gray scale rating (1 = poor–5 = excellent; ^c^ Rating for color transfer to fiber types in an attached multifiber fabric; ^d^ Color transfer after rubbing (1 = high color transfer–5 = no color transfer).

**Table 3:** Mutagenicity data for dermocybin with YG1041, TA98, TA100, TA97a, and TA1537 strains with and without S9.

Concentration (ng/µL)	Mean of Number of Revertants/Well and Standard Deviation (SD)																					
	YG1041				TA98				TA100				TA97a				TA1537				TA1537 ^a^	
	−S9		+S9		−S9		+S9		−S9		+S9		−S9		+S9		−S9		+S9		+S9	
	Mean	SD	Mean	SD	Mean	SD	Mean	SD	Mean	SD	Mean	SD	Mean	SD	Mean	SD	Mean	SD	Mean	SD	Mean	SD
Negative control	26.50	6.61	22.50	6.56	6	2.16	8	1.41	12.50	3.11	7.25	2.06	23.75	3.59	25	0.82	2	0.82	1.50	0.58	2	1.15
1.67	23.50	5.07	21.75	2.99	7.25	3.20	7.75	2.22					23	2.16	20.50	7						
4.38																	2.25	0.50	2.50	1.91	1.25	0.50
5	27	4.97	24.25	3.77	7.33	2.08	5.25	1.89	10.75	2.50	9.75	2.99	17	3.56	24	2.94						
8.75																	2.50	1.29	2	1.41	1.75	0.96
16.7	21.50	2.08	23	2.71	7.25	0.96	7	1.41	8.25	2.87	7.25	2.06	18.25	3.30	23	2.45						
17.5																	1.25	0.96	2.50	1.29	1.25	0.50
35																	1.75	1.50	2.25	1.50	1.75	0.50
50	18.50	3.11	16.75	1.50	6.75	3.30	6.50	1.91	10.75	1.50	7.75	3.86					2.75	2.87	2.75	2.22	1.25	0.50
70																	1.75	0.96	1.75	0.96	1.25	0.50
140																	1.75	1.50	2.25	1.50	1.75	0.50
167	14	3.92	10.75	3.30	5.50	2.38	8.25	3.69	10.50	1.73	7.25	4.11										
500	11	3.16	9.75	2.99	6	2.16	4.75	1.50	11	0.82	5.50	2.08	14.50	0.58	18	2.71						
Positive control ^b^	150	0	150	0	150	0	150	0	150	0	150	0	90.50	2.89	150	0	150	0	65.5	4.43	74	8.12

^a^ 10% of S9; ^b^ positive controls without metabolic activation: 4-nitro-o-phenylenediamine at 20 ng/μL for YG1041, 4-nitroquinoline-1-oxide at 1.25 ng/μL for TA98 and TA100 and 9-aminoacridine at 25 ng/μL for TA97a and TA1537. With metabolic activation: 2-aminoanthracene was used for all strains (YG1041 at 0.3 ng/μL, TA98, TA100 at 5 ng/μL, TA97a, TA1537 at 20 ng/μL).

**Table 4:** Mutagenicity data for dermorubin with YG1041, TA98, TA100, TA97a, and TA1537 strains with and without S9.

Concentration (ng/µL)	Mean of Number of Revertants/Well and Standard Deviation (SD)																					
	YG1041				TA98				TA100				TA97a				TA1537				TA1537 ^a^	
	−S9		+S9		−S9		+S9		−S9		+S9		−S9		+S9		−S9		+S9		+S9	
	Mean	SD	Mean	SD	Mean	SD	Mean	SD	Mean	SD	Mean	SD	Mean	SD	Mean	SD	Mean	SD	Mean	SD	Mean	SD
Negative control	19	3.56	22.50	6.56	5	1.41	4.50	1	9.75	2.99	8.75	2.63	21.25	2.22	24	1.83	2	0.81	1.50	0.58	2	1.15
3.33	17.25	3.30	25.75	7.27	6.50	1.29	6.50	3.11	9.50	4.20			15.50	1.29	22	6.16						
6.25																	3.25	1.70	2	1.41	1.50	1
10	17	4.90	22.50	6.56	6.75	2.36	7.75	1.71	8	2.71	8.50	1.73	17.50	1.91	14	3.37						
12.5																	1.50	0.58	2.50	0.58	1.50	0.58
25																	2.50	0.58	2.25	1.50	1.75	0.96
33.3	19.75	4.92	21.75	3.20	7.75	2.06	6.25	3.30					18.75	1.50	27	5.89						
50																	1.25	0.50	2.50	1.29	1.50	0.58
100	20.50	1	21.25	3.20	5.75	1.71	6	1.83	12.25	3.86	8	2	20.25	4.27	22.50	2.38	1.50	1	2	1.15	2.25	1.50
200																	2	1.15	3	1.63	1.25	0.50
333	21.75	3.59	24	4.76	4	1.41	7.25	2.06	12.50	1.73	9.25	5.32										
1000	20.25	4.57	22	2.31	6.25	2.50	6.25	1.71	13	2.58	10	0.82	14.26	2.06	14	1.41						
Positive control ^b^	150	0	150	0	150	0	150	0	150	0	150	0	90.50	2.89	150	0	150	0	65.50	4.43	74	8.12

^a^ 10% of S9; ^b^ positive controls without metabolic activation: 4-nitro-o-phenylenediamine at 20 ng/μL for YG1041, 4-nitroquinoline-1-oxide at 1.25 ng/μL for TA98 and TA100, and 9-aminoacridine at 25 ng/μL for TA97a and TA1537. With metabolic activation: 2-aminoanthracene was used for all strains (YG1041 at 0.3 ng/μL, TA98, TA100 at 5 ng/μL, TA97a, TA1537 at 20 ng/μL).

## Data Availability

All original data can be obtained from the authors.

## Supplementary Materials

The following supporting information can be downloaded at: https://www.mdpi.com/article/10.3390/jof8111129/s1, Table S1: HPLC conditions used in the anthraquinone purification measurements.; Figure S1: Results from the propidium iodide - digitonin assay.; Figure S2: Results from cytosolic superoxide production assay with dihydroethidium (DHE) and a general ROS production assay (DCF).

## References

[1] Yli-Heikkilä E.J., Autio M., Kylkilahti E., Räisänen R., Sekki S. (2020). Väri Materiaalina?—Kuluttajien Näkemyksiä Tekstiilien Väriaineiden Alkuperästä, Turvallisuudesta Ja Luonnollisuudesta. Kulutustutkimus.Nyt.

[2] Grand View Research (2021). Dyes & Pigments Market Size, Share & Trends Analysis Report By Product (Pigments, Dyes), By Application, By Region, And Segment Forecasts 2022-2030. https://www.grandviewresearch.com/industry-analysis/dyes-and-pigments-market.

[3] Bae J.S., Freeman H.S. (2007). Aquatic Toxicity Evaluation of New Direct Dyes to the Daphnia Magna. Dye. Pigment..

[4] de Luna L.A.V., da Silva T.H.G., Nogueira R.F.P., Kummrow F., Umbuzeiro G.A. (2014). Aquatic Toxicity of Dyes before and after Photo-Fenton Treatment. J. Hazard. Mater..

[5] Mani S., Chowdhary P., Bharagava R.N., Bharagava R.N., Chowdhary P. (2019). Textile Wastewater Dyes: Toxicity Profile and Treatment Approaches. Emerging and Eco-Friendly Approaches for Waste Management.

[6] Vacchi F.I., dos Santos A., Artal M.C., Magalhães G.R., Vendemiatti J.A.d.S., Umbuzeiro G.d.A. (2019). Parhyale Hawaiensis as a Promising Alternative Organism for Monitoring Acute Toxicity of Sediments under the Influence of Submarine Outfalls. Mar. Pollut. Bull..

[7] Korinth G., Schaller K.H., Drexler H. (2013). Percutaneous Absorption of Aromatic Amines and the Risk Assessment Resulting from the Dermal Pathway. Front. Biosci. Elit. Ed..

[8] Malinauskiene L., Bruze M., Ryberg K., Zimerson E., Isaksson M. (2013). Contact Allergy from Disperse Dyes in Textiles—A Review. Contact Dermat..

[9] Singh Z., Chadha P. (2016). Textile Industry and Occupational Cancer. J. Occup. Med. Toxicol..

[10] Fried R., Oprea I., Fleck K., Rudroff F. (2022). Biogenic Colorants in the Textile Industry—A Promising and Sustainable Alternative to Synthetic Dyes. Green Chem..

[11] Rai S., Saremi R., Sharma S., Minko S. (2021). Environment-Friendly Nanocellulose-Indigo Dyeing of Textiles. Green Chem..

[12] Venil C.K., Velmurugan P., Dufossé L., Devi P.R., Ravi A.V. (2020). Fungal Pigments: Potential Coloring Compounds for Wide Ranging Applications in Textile Dyeing. J. Fungi.

[13] Xia L., Wang A., Zhang C., Liu Y., Guo H., Ding C., Wang Y., Xu W. (2018). Environmentally Friendly Dyeing of Cotton in an Ethanol-Water Mixture with Excellent Exhaustion. Green Chem..

[14] Latos-Brozio M., Masek A. (2020). The Application of Natural Food Colorants as Indicator Substances in Intelligent Biodegradable Packaging Materials. Food Chem. Toxicol..

[15] Hunger K. (2003). Industrial Dyes.

[16] Christie R.M. (2001). Color Chemistry.

[17] Räisänen R. (2019). Fungal Colorants in Applications—Focus on *Cortinarius* Species. Color. Technol..

[18] Korulkin D., Muzychkina R. (2014). Biosynthesis and Metabolism of Anthraquinone Derivatives. World Acad. Sci. Eng. Technol. Int. J. Chem. Mol. Eng..

[19] Shukla V., Asthana S., Gupta P., Dwivedi P.D., Tripathi A., Das M. (2017). Toxicity of Naturally Occurring Anthraquinones. Adv. Mol. Toxicol..

[20] Hynninen P.H., Räisänen R., Elovaara P., Nokelainen E. (2000). Preparative Isolation of Anthraquinones from the Fungus Dermocybe Sanguined Using Enzymatic Hydrolysis by the Endogenous β-Glucosidase. Zeitschrift für Naturforsch. C.

[21] Hynninen P.H., Räisänen R. (2001). Stepwise PH-Gradient Elution for the Preparative Separation of Natural Anthraquinones by Multiple Liquid-Liquid Partition. Zeitschrift für Naturforsch. C.

[22] Brown J.P. (1980). A Review of the Genetic Effects of Naturally Occurring Flavonoids, Anthraquinones and Related Compounds. Mutat. Res. Genet. Toxicol..

[23] Sendelbach L.E. (1989). A Review of the Toxicity and Carcinogenicity of Anthraquinone Derivatives. Toxicology.

[24] Tanaka H., Morooka N., Haraikawa K., Ueno Y. (1987). Metabolic Activation of Emodin in the Reconstituted Cytochrome P-450 System of the Hepatic Microsomes of Rats. Mutat. Res. Mol. Mech. Mutagen..

[25] Jung H.A., Chung H.Y., Yokozawa T., Kim Y.C., Hyun S.K., Choi J.S. (2004). Alaternin and Emodin with Hydroxyl Radical Inhibitory and/or Scavenging Activities and Hepatoprotective Activity on Tacrine-Induced Cytotoxicity in HepG2 Cells. Arch. Pharm. Res..

[26] Bach E., Cleve E., Schollmeyer E. (2002). Past, Present and Future of Supercritical Fluid Dyeing Technology—An Overview. Color. Technol..

[27] Räisänen R., Montero G.A., Freeman H.S. (2021). A Fungal-based Anthraquinone Emodin for Polylactide and Polyethylene Terephthalate in Supercritical Carbon Dioxide (SC-CO2) Dyeing. Color Res. Appl..

[28] Saus W., Knittel D., Schollmeyer E. (1993). Dyeing of Textiles in Supercritical Carbon Dioxide. Text. Res. J..

[29] Hou A., Chen B., Dai J., Zhang K. (2010). Using Supercritical Carbon Dioxide as Solvent to Replace Water in Polyethylene Terephthalate (PET) Fabric Dyeing Procedures. J. Clean. Prod..

[30] Räisänen R., Nousiainen P., Hynninen P.H. (2001). Emodin and Dermocybin Natural Anthraquinones as High-Temperature Disperse Dyes for Polyester and Polyamide. Text. Res. J..

[31] American Association of Textile Chemists and Colorists (AATCC) (2016). AATCC TM8 – Test Method for Colorfastness to Crocking: Crockmeter Method.

[32] American Association of Textile Chemists and Colorists (AATCC) (2013). AATCC TM61–Test Method for Colorfastness to Laundering: Accelerated.

[33] Pesonen M., Pasanen M., Loikkanen J., Naukkarinen A., Hemmilä M., Seulanto H., Kuitunen T., Vähäkangas K. (2012). Chloropicrin Induces Endoplasmic Reticulum Stress in Human Retinal Pigment Epithelial Cells. Toxicol. Lett..

[34] Vellonen K.-S., Honkakoski P., Urtti A. (2004). Substrates and Inhibitors of Efflux Proteins Interfere with the MTT Assay in Cells and May Lead to Underestimation of Drug Toxicity. Eur. J. Pharm. Sci. Off. J. Eur. Fed. Pharm. Sci..

[35] Loikkanen J.J., Naarala J., Savolainen K.M. (1998). Modification of Glutamate-Induced Oxidative Stress by Lead: The Role of Extracellular Calcium. Free Radic. Biol. Med..

[36] Höytö A., Herrala M., Luukkonen J., Juutilainen J., Naarala J. (2017). Cellular Detection of 50 Hz Magnetic Fields and Weak Blue Light: Effects on Superoxide Levels and Genotoxicity. Int. J. Radiat. Biol..

[37] OECD (2018). Test No. 442D: In Vitro Skin Sensitisation.

[38] Zwarg J.R.R.M., Morales D.A., Maselli B.S., Brack W., Umbuzeiro G.A. (2018). Miniaturization of the Microsuspension *Salmonella*/Microsome Assay in Agar Microplates. Environ. Mol. Mutagen..

[39] Umbuzeiro G., Morales D., Vacchi F., Albuquerque A., Szymczyk M., Sui X., Vinueza N., Freeman H. (2021). A Promising Ames Battery for Mutagenicity Characterization of New Dyes. Environ. Mol. Mutagen..

[40] Brown J.P., Brown R.J. (1976). Mutagenesis by 9,10-Anthraquinone Derivatives and Related Compounds in *Salmonella Typhimurium*. Mutat. Res. Toxicol..

[41] Mortelmans K., Zeiger E. (2000). The Ames *Salmonella*/Microsome Mutagenicity Assay. Mutat. Res. Mol. Mech. Mutagen..

[42] Freeman H.S. (2022).

[43] Räisänen R., Björk H., Hynninen P.H. (2000). Two-Dimensional TLC Separation and Mass Spectrometric Identification of Anthraquinones Isolated from the Fungus Dermocybe Sanguinea. Zeitschrift für Naturforsch. C.

[44] Danielsen K., Aksnes D.W., Francis G.W. (1992). NMR study of some anthraquinones from rhubarb. Magn. Reason. Chem..

[45] Draper S., Montero G., Smith B., Beck K. (2000). Solubility Relationships for Disperse Dyes in Supercritical Carbon Dioxide. Dye. Pigment..

[46] Panigrahi G.K., Yadav A., Srivastava A., Tripathi A., Raisuddin S., Das M. (2015). Mechanism of Rhein-Induced Apoptosis in Rat Primary Hepatocytes: Beneficial Effect of Cyclosporine A. Chem. Res. Toxicol..

[47] Guo C., Sun L., Chen X., Zhang D. (2013). Oxidative Stress, Mitochondrial Damage and Neurodegenerative Diseases. Neural Regen. Res..

[48] Ott M., Gogvadze V., Orrenius S., Zhivotovsky B. (2007). Mitochondria, Oxidative Stress and Cell Death. Apoptosis.

[49] Singh J., Hussain Y., Luqman S., Meena A. (2021). Purpurin: A Natural Anthraquinone with Multifaceted Pharmacological Activities. Phyther. Res..

[50] Griesinger C., Casati S., Worth A., Whelan M. (2014). EURL ECVAM Recommendation on the KeratinoSensTM Assay for Skin Sensitisation Testing.

[51] Kim S.-H., Lee D.H., Lee J.H., Yang J.-Y., Shin H.-S., Lee J., Jung K., Jeong J., Oh J.-H., Lee J.K. (2020). Evaluation of the Skin Sensitization Potential of Carbon Nanotubes Using Alternative *In Vitro* and *In Vivo* Assays. Toxics.

