# Supplementary material for: Waterless Dyeing and In Vitro Toxicological Properties of Biocolorants from Cortinarius sanguineus
Source: J Fungi (Basel). 2022 Oct 26;8(11):1129. doi: 10.3390/jof8111129 (PMC9694584; doi:10.3390/jof8111129)
Supplement: Supplementary file 1 [file jof-08-01129-s001.zip › jof-1947054-supplementary.pdf]

Journal of
Fungi

MDPI

Supplementary Materials

Table S1. HPLC conditions used in the anthraquinone purification measurements.

Time (min)		Acetonitrile (%)	0.1% Formic Acid (%)
15 Min Run Time	30 Min Run Time		
0	0	48	52
3	3	70	30
10	25	70	30
15	30	48	48

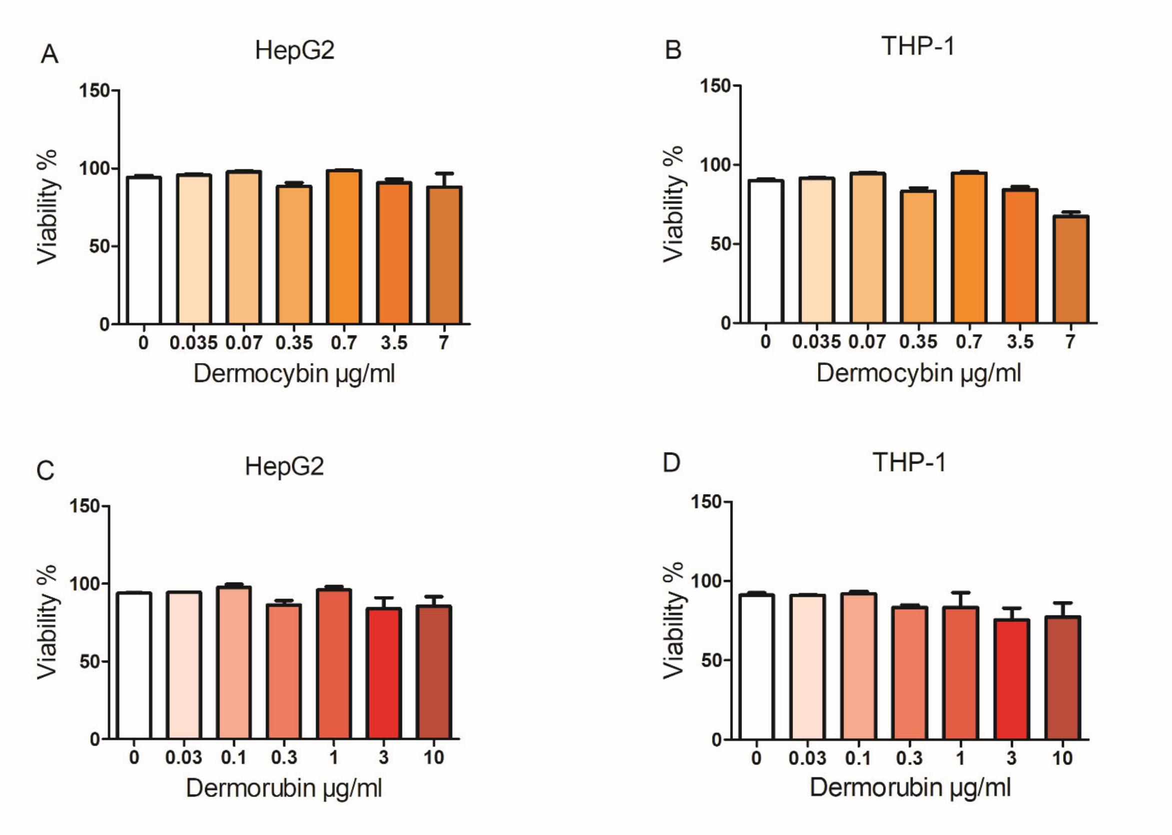

Figure S1. Results from the propidium iodide - digitonin assay. The viability is shown as mean ± SEM. No statistically significant differences were observed. Figures (**A**) and (**B**) depict the results obtained with dermocybin in HepG2 and THP cell lines, and figures (**C**–**D**) show the corresponding experiments using dermorubin. $n = 3$.

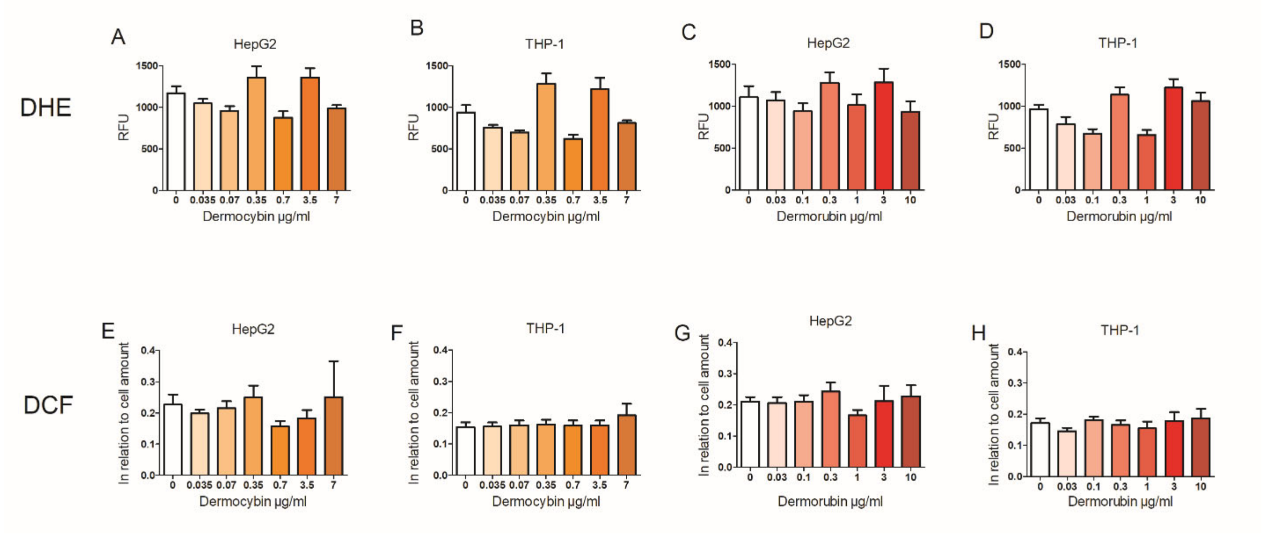

Figure S2. Results from cytosolic superoxide production assay with dihydroethidium (DHE) and a general ROS production assay (DCF). Figures (**A**–**D**) show the results from the DHE assay conducted using dermocybin or dermorubin as relative fluorescence units. Data is shown as fluorescence mean ± SEM. Figures (**E**–**H**) show the results from the DCF Assay. Data is shown mean ± SEM, *n* = 3 in all experiments.
